# The Contribution of Cultural Heritage Owned by Local Health Authorities in the Humanization of Care: The Point of View of Top Management

**DOI:** 10.3390/ijerph192416632

**Published:** 2022-12-11

**Authors:** Martina Giusti, Claudia Cosma, Stefania Simoni, Niccolò Persiani

**Affiliations:** 1 Department of Experimental and Clinical Medicine, University of Florence, 50134 Florence, Italy; 2 Department of Health Science, University of Florence, 50134 Florence, Italy

**Keywords:** patient, health impact assessment, public health resources, historical hospitals

## Abstract

After the COVID-19 pandemic, reforms in healthcare systems have the purpose to fully recover the relationship of healthcare organizations with their patients. For centuries, art was used throughout Europe in the healthcare context for its power to engage and support patients in their illnesses. This approach can be rediscovered by utilizing the cultural heritage owned by Local Health Authorities. In this context, tradition, art, innovation, and care coexist. This study aims to investigate the interest in developing projects for the humanization of care by the top management of Italian Local Health Authorities, in particular exploiting their cultural heritage. The evaluation of the proposal was conducted using semi-structured interviews with the top management of two Local Health Authorities, in which the Santa Maria Nuova hospital in Florence and the Santo Spirito in Sassia Hospital in Rome are located, as the two selected cases for this study. The interviewees welcomed the proposal to develop humanization of care projects involving the use of their cultural heritage. Moreover, they expressed their desire to invest human, economic, and structural resources in the development of these initiatives. The implementation of humanization of care projects using cultural heritage owned by Local Health Authorities is useful to apply specific policies to enhance the governance of the cultural heritage according to the health mission. On the other hand, it permits the search for additional or ad hoc resources. Finally, it is possible to humanize and improve patients’ experience while increasing awareness among the health workforce and trainees.

## 1. Introduction

Health organizations should always guarantee the humanization of care. It must be true also when isolation and lockdowns are imposed at either local or worldwide level, such as during the COVID-19 pandemic. The introduction of physical barriers to minimize the risk of infection and the limitation of personal freedom led to a reduction in human interactions during the COVID-19 pandemic. As such, the relationship between healthcare workers and patients should be restored [1]. This can be done through the rediscovery and the new application of patient-centred care approach.

Since the 1960s, patient-centred care has been continuously reproposed as the main solution to fight the disease-oriented model of medicine. It supposes that patients are the protagonists of their own care pathway. Patient-centred care is, in fact, a personalized approach that considers patients’ attitudes and guarantees the self-determination of patients [2,3]. In daily clinical practice, patient-centred care integrates relational and psychological aspects through the collaboration between practitioners, patients, and, possibly, caregivers [4]. So, patient-centred care is based on the humanization of care. 

Humanization of care means putting the human person at the centre of health and social care pathways in a holistic view, i.e., considering the inseparable totality of the physical, mental, emotional, and spiritual components [5]. 

Among various models of humanization of care, those using art have been mostly successful [6]. Art has always played an important leading role in hospitals for its intrinsic capacity to bring closer strangers, indigents, or patients. Art helps to deal with the condition of physical, mental, or economic fragility [7]. 

Between the IV and XII centuries, art inspired by Christianity was used in hospitals to provide comfort and hope to alleviate the suffering of patients [8]. When health care activities became gradually predominant over hospitality, art changed its mission. Art accompanied patients in the health care pathway, helping them to maintain their good psycho-physical condition, in addition to the responsiveness to provided health treatments [9]. 

After the second half of the Middle Ages (12th–13th century), hospitals became secular institutions following the progressive affirmation of critical and rational thinking. Hospital art ceased to be an instrument of evangelization and humanization of care and became instead a tool of personal affirmation for the commissioners of the artworks [10]. As the relationship between art and health disappeared as a subsidiary element of providing care, cultural heritage started to represent and be identified with the society it belonged to [11]. 

Nowadays, the link between art and health has been rediscovered through two distinct manners. The first approach sees art as a structural element of hospitals, being instrumental to overcome the idea that these places are aseptic and depersonalized [12]. Art supports patient-centred care through humanizing the relationships between the health settings and the patients. The second way consists of art therapy [13]. Art becomes a proper therapeutic support tool in multiple care contexts, ranging from intensive care unit [14] to mental health departments [15] and outpatient care [16]. The World Health Organisation (WHO) has also recognized the contribution of art to the promotion of mental and physical well-being and healthy lifestyles, as well as to the correct management of a care pathway [17]. 

The role of art in any healthcare organisations is defined by the vision of the Local Health Authority top management. The integration of art in healthcare settings is effective if the top management of health care organizations sets it as a strategic objective [18]. Top management can adopt health policies promoting the humanization of care with the use of art [19]. To do this, it is necessary to sensibilize, raise awareness, and provide training to healthcare personnel [20]. Obviously, this requires the allocation of specific structural, personnel, and economic-financial resources [21,22]. 

In the literature, no studies have considered the possibility of using the cultural heritage owned by Local health Authorities for the humanization of care. Therefore, this paper aims to fill this gap by investigating the willingness of the top management of healthcare organizations to develop projects for the humanization of care that can exploit the cultural heritage in their possession.

## 2. Materials and Methods

The governance adopted by four Italian Health Authorities for the management of their own cultural heritage was compared in 2019 [23]. The qualitative approach was identified as the appropriate method for conducting this type of research [24]. 

Among the four case studies, the Azienda USL Toscana Centro and the ASL Roma 1 were selected as significant cases for three reasons. The first reason is the presence of a strong collaboration between administrative and health management in the development of health policies. It is useful to describe and compare the different points of views of all top management on the possible use of cultural heritage in the implementation of projects of humanization of care. The second reason is the presence of a historical hospital where a rich cultural heritage coexists with a modern and technological health organization: the Santa Maria Nuova hospital in Florence, operating under the Azienda USL Toscana Centro, and the Santo Spirito in Sassia hospital in Rome, operating under the ASL Roma 1. Finally, the third reason is the implementation of ad hoc governance models for the protection and enhancement of their own cultural heritage. 

After identifying the cases for this study, the research group conducted semi-structured interviews to analyse and compare the perspectives of the top management of these Local health Authorities about the proposal of implementing humanization of care projects using their own cultural heritage. Contextually, there was also the desk analysis of documents related to the models of governance and management of the cultural heritage adopted by considered Local Health Authorities. 

The general managers of the Local Health Authorities were included in the sample as the main decision-makers of corporate health policies. The cultural heritage managers were engaged as the promoters and the coordinators of possible initiatives, that could provide the use of cultural heritage in humanization of care projects. Finally, the health managers were involved as the organizers of specific care pathways and promoters of training course for health professionals.

Potential participants were contacted by emails explaining the purpose of the research and listing the main topics of the interview. The managers, who decided to took part in this study, were contacted again to determine the date and the most useful method of conducting the interview (online or face-to-face). Between June and September 2022, a team of 3 researchers conducted 6 semi-structured interviews.

1. Four questions were asked to fill the knowledge gaps identified in the literature regarding the specific projects of humanization of care.

The interviews were recorded, after obtaining the oral consent of the respondents. 

After the interviews were transcribed, the responses were coded and analysed. Four coding categories were developed according to the identified gaps in the literature: (i) use of cultural heritage in the humanization of care; (ii) allocation of specific resources for financing humanization of care projects; (iii) training of health personnel; and (iv) target identification for humanization of care pilot projects. Through this coding stage, the main suggestions offered by the top management personnel were identified. 

**Figure 1:**
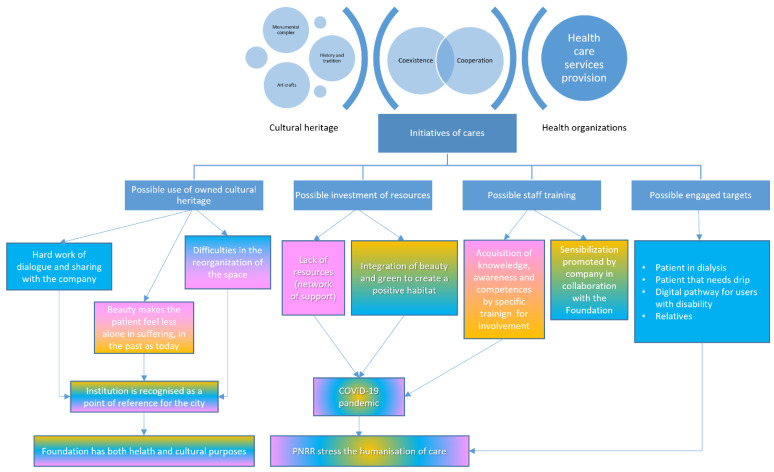
Coding process illustrating the results of the Santa Maria Nuova hospital case (general management in yellow, cultural heritage management in blue, health management in pink).

**Figure 2:**
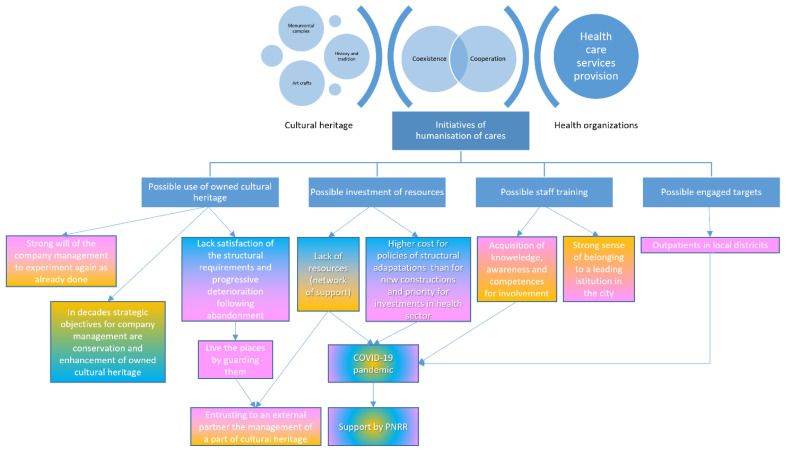
Coding process illustrating the results of the Santo Spirito in Sassia hospital case (general management in yellow, cultural heritage management in blue, health management in pink).

An information tree was constructed for each case in accordance with the coding logic. The different contributions coming from different categories of management personnel are integrated and highlighted in Figure 1 and in Figure 2 by colour: yellow for replies from general management, blue for replies from heritage management, and pink for replies from health management. The two information trees were directly compared to identifying the similarities and differences between two cases. 

## 3. Case Studies

### 3.1. The Santa Maria Nuova Hospital and the Santa Maria Nuova Foundation Onlus

The Santa Maria Nuova hospital is the oldest working hospital in the world, which is still operating on the same original site. It was built in 1288 by the order of Folco Portinari, a rich trader at that time. He wanted to donate a hospital to Florence for sustaining the development of the city. Folco Portinari was famous as the father of Beatrice Portinari, with whom Dante Alighieri fell in love.

The Santa Maria Nuova hospital expanded itself over the centuries thanks to fundings from nobles, kings, and Popes, who recognized the contribution of this institution to the well-being and growth of the city. The Santa Maria Nuova hospital was able to engage some of the most famous artists of the Renaissance in Florence, such as Ghiberti and Buontalenti. Artists adorned the hospital space and inhabited it, populating its porticoes [25]. 

The Santa Maria Nuova hospital is a fantastic example of art and healthcare coexistence. Thanks to the complete restructuration of the building (from 1999 to 2005 and financed by Tuscany Region with the investment of about 24.5 million in Euro), the Santa Maria Nuova hospital is currently a modern hospital in a Medieval building. The ancient structure was renovated in compliance with the latest international structural and technological standards, quality care measures, safety index and comfort markets for patients and workers. Since 1288, the hospital has always been able to adapt itself to the continuous progress in the field of health and clinical assistance. 

The importance of preserving, enhancing, and improving the use of their own cultural heritage prompted the management of the Azienda USL Toscana Centro to set up the ‘Santa Maria Nuova Foundation Onlus’ in 2015. The Azienda USL Toscana Centro retains the property of the cultural heritage, while its management is entrusted to the Foundation. At first, the jurisdiction of the Foundation was only the Santa Maria Nuova hospital. Since 2020, its authority has extended to all 13 hospitals of the Azienda USL Toscana Centro. The main purposes of the Foundation are to maintain and to promote the cultural heritage using a structured museum itinerary. The Foundation uses their own resources, especially donations, to sustain these activities [26].

### 3.2. The Hospital Santo Spirito in Sassia and Its Monumental Complex

The Santo Spirito in Sassia Hospital in Rome was founded in 727 A.D. by Sasson king Ina as a hostel for Saxon pilgrims. 

In the 13th century, Pope Innocenzo III decided to transform the hostel, which was destroyed by a fire, into a facility to support poor and suffering people and to welcome abandoned infants [27]. Thus, the hospital was built. 

The hospital continued its activities until 1471, when it was devastated once again by a massive fire. Pope Sisto IV (1471–1484) visited the hospital shortly after his enthronement and ordered its immediate reconstruction for the Jubilee.

In the 16th century, the hospital stood out as a place of innovation, beauty, and holiness known across all Europe. Artists, scientists, and religious figures, such as St. Filippo Neri, St. Camillo De Lellis, Michelangelo, Leonardo Da Vinci, and Botticelli, were hosted here during their time in Rome. 

Over the centuries, the Hospital of Santo Spirito in Sassia was protected by the Popes, who made considerable donations. These resources were used to sustain its charitable mission, its scientific research, and the institution’s prestige by the enrichment of its artistic wealth [28,29].

In 1896, the Santo Spirito in Sassia Hospital was the main hospital in Rome, as well as the largest hospital in Europe, with more than 15,000 hectares of land holdings. This wealth was the result of the union of the Santo Spirito Hospital with minor Roman hospitals under the name of “Pio Istituto di Santo Spirito ed Ospedali Riuniti di Roma”. 

In 1978, the establishment of the National Health System in Italy led to the establishment of the current Local Health Authority. 

Today, the Santo Spirito in Sassia Hospital is a part of ASL Roma 1, a public institution created on 1 January 2016 by the merging the ASL Roma A and the ASL Roma E. It is part of an extended monument complex, which includes active hospitals, a church, libraries, ancient buildings, a museum, outpatient clinics, and gardens. 

The Local Health Authority of the ASL Roma 1 is responsible for guaranteeing a satisfactory health service for the affiliated population, with its resources coming from the State from the funding of the National Healthcare System. At the same time, these resources are used to maintain and valorize the owned cultural heritage that is directly managed by the office for patrimony of the Asl Roma 1.

## 4. Results

During the interviews, all six interviewees showed a strong interest in experimenting humanization of care through the cultural heritage owned by their Local Health Authorities. The interviewed managers made themselves available to be the first promoters for the implementation of this kind of projects in reality. The positive impacts in terms of improving health care quality and exploiting the possessed cultural heritage were discussed by the interviewees.

The top management of the Azienda USL Toscana Centro (Figure 1) highlighted the important role played by ‘Fondazione Santa Maria Nuova Onlus’ in the cultural heritage governance. 

As the general secretary of the Foundation, Dr. C. B represents both the developer and the promoter of initiatives that take advantage of the cultural heritage. Thanks to her profound knowledge of the managed cultural heritage, she proposed projects to integrate the management of the cultural heritage with health activities, especially in relation to the humanization of care. This is also one of the strategic objectives that the Santa Maria Nuova Foundation Onlus included in their new Statute. The activities of cultural heritage protection and valorization are joined by those that promote individual and collective health. For this reason, she has constantly invited health professionals to participate in this kind of projects. 

The general management, represented here by the delegate of the general manager Dr. G.L. (Director of the Department of Specialized Medical Disciplines of the Azienda USL Toscana Centro), reiterated the strategic importance of the Santa Maria Nuova Foundation Onlus creation in 2015. Indeed, the Foundation favours the integration of initiatives both of the health care promotion and of cultural heritage valorisation.

The health management of the Santa Maria Nuova hospital, as represented by Dr. E.C. (Health Director of the Santa Maria Nuova hospital), empathized the supporting role of art in the proposed projects. However, she also underlined the difficulties to use the cultural heritage in social and health care activities for the constraints imposed for its maintenance. Nevertheless, the value of the proposal and the need to act in order to ensure its success were recognized. This is especially true at the present time. The COVID-19 pandemic has highlighted the importance of humanization of care and the relevance of developing patient-centred care pathways. This is possible by starting with the proposed projects of using art to personalize and humanize the health sector. 

In the ASL Roma 1 (Figure 2), the close collaboration between the general management (i.e., Dr. A.T., general director of the ASL Roma 1) and the health management (i.e., Dr. P.C., director of the Area of Hospital Management Area and health director of the Santo Spirito in Sassia Hospital) leads to an effective, shared, and consolidated strategy that aims to protect and valorise the cultural heritage owned by the Local Health Authority. Both levels of management recognized the added value of using their own cultural heritage to humanize care and promote related initiatives. Through their collaboration, they can optimize the use of their cultural heritage in economic and health terms. However, the general and health management suggested different ways of reaching the shared goals. While the general management sustained the entrustment of the cultural heritage governance to an external partner, the health management preferred an internal one. 

The Italian National Recovery and Resilience Plan was developed to overcome the economic crisis due to the COVID-19 pandemic. In the plan’s mission 6 “Health”, resources can be invested in the requalification of ancient and historical buildings (i.e., monumental complexes, churches, and so on). It is an opportunity that has never presented itself before and can be fully exploited. The use of cultural heritage in the humanization of care can be a driver of specific investments for the restoration and promotion of properties. 

Finally, the cultural heritage management (i.e., Dr. F.B., director of the Technical Department of the ASL Roma 1 heritage office) emphasized its strategic role as the guarantor of the maintenance and management of cultural heritage. Nevertheless, the corporate strategy needs to be review. Clear objectives and unambiguous implementation roadmaps must be developed to satisfy the emerging issues about the use, maintenance, and promotion of possessed cultural heritage. 

Comparing the two selected cases, the adoption of different governance strategies follows the need to respond to different problems, even though the final objectives are the same. 

The ASL Roma 1 shows the results of a current lack of resources to be invested in the restoration and structural adaptation of historic buildings. This is linked to the constraints posed on the use of resources allocated for the provision of health care services. 

In the Azienda USL Toscana Centro, the focus is on the implementation of a medium-long plan of cultural heritage promotion to maximize the exploitation of existing investments. In fact, the Santa Maria Nuova hospital was completely restored and refurbished at the beginning of the 1990s. The scope is to adapt the original building to the current technological and structural standards. Another significant aspect is the relationship between owners and managers of its cultural heritage. The Santa Maria Nuova Foundation Onlus and the Azienda USL Toscana Centro operate on parallel but distinct paths. Thus, it is difficult to establish a close dialogue, although the collaboration between the entities is constant and fruitful. The presence of the Foundation has led both the administrative and health management to concentrate exclusively on pursuing health objectives. In fact, the Foundation has been completely entrusted to manage, value, and use the cultural heritage, including in humanization of care projects. 

Instead, there is a strong synergy between the administrative and health departments and the heritage office in the Asl Roma 1. All jointly work toward the development and promotion of initiatives for the humanization of care, defining a shared strategy to compensate the absence of a dedicated governance for the cultural heritage. Together, they validate the choice made by Azienda USL Toscana Centro to rely on external institutions for the management of the cultural heritage. In fact, the Florentine experience shows how this governance model simplifies the management of cultural heritage and overcomes the bureaucratic constrains of public administrations. However, on the other hand, it can also lead to a major disconnection between the maintenance and enhancement of cultural heritage and the provision of healthcare services. 

All respondents agreed that the training of the health workforce is a crucial step toward the implementation of humanization of care projects. Indeed, health professionals are meant to be the main creators and promoters of humanization of care in clinical settings. Therefore, they should be sensitized and trained on the use of the cultural heritage owned by their Local Health Authorities in these pathways. 

The impact of humanization of care projects using cultural heritage is limited, if these projects are offered directly to users. To guarantee their effectiveness, these projects have to be rooted in the day-to-day activities of the Local Health Authorities and managed directly by health professional.

Outpatients, patients with chronic diseases under control, patients in periodic treatments, and hospitalized patients with possibility of moving were identified as the best target groups to be primarily involved in the humanization of care projects. Population targets involved in prevention and health educational campaigns or in screening may be also considered.

## 5. Discussion

For all interviewees, the use of the cultural heritage possessed by local health organizations can contribute toward the humanization of care projects. Nevertheless, they highlighted a series of difficulties connected to the realization of these initiatives. 

The main problem is to carry out healthcare activities in a setting of historical and cultural relevance. It is particularly complex to adapt and use these spaces so that they adhere to current structural and organizational requirements. Here, works of art and cultural heritage assets are a living testimony of the history and the culture of these institutions, which have passed down to us through the centuries. 

The entrustment of the cultural heritage owned by the analyzed Local Health Authorities to external private partners appears an agreeable solution for its protection and valorization. Nevertheless, the organizations want to maintain their full ownership. 

In Florence, this model is already working, and the ‘Fondazione Onlus Santa Maria Nuova’ is already identified as a reference partner by the Azienda. 

In Rome, the concession of the cultural heritage management to an external partner is under evaluation. At the same time, the top management of the ASL Roma 1 also takes into account the good results obtained by the consolidated inter-institutional network for the management of its cultural heritage (municipality of Rome, Capitoline supervision of the cultural heritage, Italian Ministry of Culture, other Italian Local Health Authorities, etc). In terms of the humanization of care, the network has already experienced it. In fact, the use of art in social and health context is a reality in the ASL Roma 1. For years, art therapy has been adopted in multiple settings and clinical pathways [16,17,18,19,20]. In the museum “Laboratory of Mind” of the ASL Roma 1 (member of the network “Menti in rete”), art is also used as an effective communicative tool in campaigns of sensibilization about mental health. The most critical issues of this model are always about finding ad hoc economic and human resources for the conservation, valorisation, and realization of cultural heritage and the regulatory and bureaucratic constraints of public organizations. 

The entrustment of cultural heritage to an external partner makes it possible to overcome these limitations, which can impede the research on economic resources and specific expertise (e.g., art historians, restorers, and marketing experts) necessary for the development of effective policies for cultural heritage management. 

This is also affecting the opportunities for the integration of different cultural heritage promotion initiatives within the health objectives of the cultural heritage owners. The Santa Maria Nuova Foundation Onlus is a fantastic example.

All the interviewed professionals agreed that a renewal of the National Healthcare System after the COVID-19 pandemic starts with the humanization of care projects, including by using art. In fact, the aim of humanization of care is to enforce the relationship between patients and the healthcare system. This path began in the 1960s and is currently at a turning point [4]. The disease-oriented medical approach was extremely stressed during the emergency phase of the COVID-19 pandemic, when resources were exclusively allocated to the treatment of the pathology. In the last two years, this phenomenon has contributed to a growing awareness of the importance of operatively implementing humanization of care projects and patient-centred care [2,3,4]. 

To do this, training programs about humanization of care and application techniques for health professionals should be organized [30]. For all respondents, the engagement of health professionals should be the first step to implement the projects for the humanization of care, especially exploiting the cultural heritage of the organizations. Since health professionals are in direct contact with patients and their caregivers, they are the first promoters of these new projects. Health professionals also define the clinical pathways. They might be the best personnel to introduce art as therapeutic support. In addition, they should be effectively informed about the positive impact of art on patients’ mental well-being and their response to treatments [17].

The two studied cases have already launched initiatives to raise awareness among health professionals of the use of art in healthcare through workshops, seminars, and lectures. Furthermore, health professionals can be directly engaged in the experimentation of cultural heritage use in these humanization of care projects. 

The current reform of the Italian National Health System due to the National Recovery and Resilience Plan strengthens the management of patients at home or at local level. In this regard, the health facilities within the reference territory of a Local Health Authority are subjected to recovery and enhancement interventions by recovery and preservation actions. Contextually, projects of humanization of care using cultural heritage are welcomed because they enable the reinforcement of the relationship between patients and the health institutions. 

From this perspective, the Azienda USL Toscana Centro would integrate health objectives with cultural ones in local health facilities, with a minor level of complexity. Here, in fact, it would be easier to develop a stronger sense of belonging both in users and in health professionals. This direction is also evident from the positive experience of the ASL Roma 1. In the diffuse monumental complex of the Santo Spirito in Sassia Hospital, some initiatives of art therapy and humanization of care through its own cultural heritage have been attempted. The most relevant experience is the museum “Laboratory of Mind”, where art is used as an optimal means of communication in projects of primary prevention or wellness and well-being promotion in mental health. Another fantastic example is the assistance offered to patients with dependence or age-related/senile cognitive impairment in cloisters, gardens, libraries, and museums. In the clinical pathways of these patients, art therapy is included as a treatment support [16,17].

The interviewees did not indicate specific targets for the pilot projects to trial the humanization of care using cultural heritage. In the literature, a lot of studies have evidenced the benefits of humanization of care using cultural heritage for the elderly [31], children [32], caregivers [33], and persons with mental health disorders [34,35]. Maybe these patients might be the first ones to be involved in future projects. However, it is preferable that they are outpatients, patients with cyclical treatments, or hospitalized patients to minimize organizational and logistic problems. The execution of visits in the gardens instead of in the clinic and the provision of outpatient treatments in monumental areas of historical hospitals were suggested as possible interventions to start implementing the humanization of care using the cultural heritage owned by Local Health Authorities. For hospitalized patients, the creation of digital museum routes made available via tablets was proposed. 

Finally, our interviewees seemed to be really interested in the activation of humanization of care projects for the positive impact at the organizational and management level [36]. According to their possibilities, the top management may orient to and favour these paths and monitor this kind of benefits. As mentioned above, the first step is the involvement and training of health professional [37]. They should know that the introduction of humanization of care projects through cultural heritage will improve not only the organization’s performance or the satisfaction of patients, but also their ways of working. Patients should perceive a higher quality in the provision of health services which, in turn, will improve their compliance. This new type of healthcare should also improve patients’ response to treatments, by decreasing their levels of stress and anxiety. In addition, health professionals could operate in a more relaxed and serene environment, taking advantage of a better organizational environment. 

## 6. Conclusions

The top management of the Azienda USL Toscana Centro and the ASL Roma 1 expressed the willingness of their healthcare organizations to support the development of projects for the humanization of care. They want to invest structural, economic, and professional resources in the implementation of projects for the humanization of care using cultural heritage owned by Local Health Authorities, albeit in compliance with the present regulatory constraints. They also agreed that the first step is the specific training of healthcare personnel. Training courses about using art in the humanization of care could be held as part of the lifelong education of health professionals in collaboration with the training offices of the two Local Health Authorities considered in this study. Health professionals could learn techniques and operational tools to use to humanize their activities, while also exploiting the cultural heritage. Universities could also be involved in the training of future health professionals on the humanization of care. Existing modules on ethics, deontology, and communication could be enriched with a focus on this specific topic. 

The effective engagement of trained health professionals is fundamental. In the first instance, health professionals are the first promoters and actuators of the humanization of care using the cultural heritage of their Local Health Authority. In the second instance, they might guide patients and their caregivers into experimenting this new type of support, informing, and educating them. Finally, health professionals have the expertise to identify targets that would gain the highest benefits from this type of support. 

Therefore, the top management of Local Health Authorities have to promote policies which sustain experimentations, workshops, or projects about the humanization of care using cultural heritage. For example, premiums can be introduced for health professionals to be involved in these initiatives. 

## Data Availability

The data presented in the study are available from the corresponding author upon request. The data are not publicly available due the privacy of interviewed respondents.

## References

[1] D’Ambrosio F., de Belvis A.G., Morsella A., Castellini G., Graffigna G., Laurenti P. (2020). Life after COVID-19: Rethinking the Healthcare System and Valuing the Role of Citizens’ Engagement in Health Prevention. Front. Psychol..

[2] Danis M., Solomon M. (2013). Providers, Payers, the Community, and Patients Are All Obliged to Get Patient Activation and Engagement Ethically Right. Health Aff. (Proj. Hope).

[3] Schmutte T., Davidson L., O’Connell M. (2018). Improved Sleep, Diet, and Exercise in Adults with Serious Mental Illness: Results from a Pilot Self-Management Intervention. Psychiatr. Q..

[4] Gusmano M.K., Maschke K.J., Solomon M.Z. (2019). Patient-Centered Care, Yes; Patients as Consumers, No. Health Aff..

[5] Busch I.M., Moretti F., Travaini G., Wu A.W., Rimondini M. (2019). Humanization of Care: Key Elements Identified by Patients, Caregivers, and Healthcare Providers. A Systematic Review. Patient.

[6] Boyce M., Bungay H., Munn-Giddings C., Wilson C. (2018). The Impact of the Arts in Healthcare on Patients and Service Users: A Critical Review. Health Soc. Care Community.

[7] Peggy J., Griffin Donald J. (2010). History of hospitals and health care. Hospitals: What They Are and How They Work.

[8] Delamothe T. (1989). Hospital Art and Its Problems. BMJ (Clin. Res. Ed.).

[9] Baron J.H. (1995). Art in hospitals: The Fitzpatrick Lecture 1994. J. R. Coll. Physicians Lond..

[10] Murzyn-Kupisz M., Działek J. (2013). Cultural heritage in building and enhancing social capital. J. Cult. Herit. Manag. Sustain. Dev..

[11] Staricoff R.L. (2004). Arts in Health: A Review of the Medical Literature.

[12] Macnaughton J. (2007). Art in hospital spaces: The role of hospitals in an aestheticised society. Int. J. Cult. Policy.

[13] Malchiodi C.A. (2011). Handbook of Art Therapy.

[14] Shella T.A. (2018). Art therapy improves mood, and reduces pain and anxiety when offered at bedside during acute hospital treatment. Arts Psychother..

[15] Leckey J. (2011). The therapeutic effectiveness of creative activities on mental well-being: A systematic review of the literature. J. Psychiatr. Ment. Health Nurs..

[16] Zuo X., Lou P., Zhu Y., Chen B., Zhu X., Chen P., Dong Z., Zhu X., Li T., Zhang P. (2022). Effects of expressive art therapy on health status of patients with chronic obstructive pulmonary disease: A community-based cluster randomized controlled trial. Ther. Adv. Respir. Dis..

[17] Fancourt D., Finn S. (2019). What Is the Evidence on the Role of the Arts in Improving Health and Well-Being? A Scoping Review.

[18] Bokhour B.G., Fix G.M., Mueller N.M., Barker A.M., Lavela S.L., Hill J.N., Solomon J.L., Lukas C.V. (2018). How can healthcare organizations implement patient-centered care? Examining a large-scale cultural transformation. BMC Health Serv. Res..

[19] Berkowitz B. (2016). The patient experience and patient satisfaction: Measurement of a complex dynamic. Online J. Issues Nurs..

[20] Sikka R., Morath J.M., Leape L. (2015). The quadruple aim: Care, health, cost and meaning in work. BMJ Qual. Saf..

[21] Baron J.H., Greene L. (1984). Art in hospitals. Funding works of art in new hospitals. Br. Med. J. (Clin. Res. Ed.).

[22] Youngson R., Blennerhassett M. (2016). Humanising healthcare. BMJ.

[23] Galimberti P.M. (2022). Lo Splendore della Cura: Viaggio Negli Ospedali Storici d’Italia.

[24] Dion D. (1998). Evidence and inference in the comparative case study. Comp. Politics.

[25] Diana E. (2012). Santa Maria Nuova, Ospedale dei Fiorentini: Architettura ed Assistenza Nella Firenze tra Settecento e Novecento.

[26] Landini G. (2017). Santa Maria Nuova Attraverso i Secoli: Assistenza, Scienza e Arte nell’ospedale dei Fiorentini.

[27] Colonna F. (2009). L’Ospedale di Santo Spirito a Roma: Lo Sviluppo dell’assistenza e le Trasformazioni Architettonico-Funzionali.

[28] Mattoni S., Scarnò M., Valensise M.R., Mongardini M., Bucci R. (2012). From a Pope’s nightmare, a great public health institution: The Santo Spirito in Saxia Hospital, in Rome. Ital. J. Public Health.

[29] Presciutti D.B. (2011). Dead infants, cruel mothers, and heroic popes: The visual rhetoric of foundling care at the hospital of Santo Spirito, Rome. Renaiss. Q..

[30] Schmidt H. (1998). Integrating the Teaching of Basic Sciences, Clinical Sciences, and Biopsychosocial Issues. Acad. Med. J. Assoc. Am. Med. Coll..

[31] Melo R.C.C.P.D., Costa P.J., Henriques L.V.L., Tanaka L.H., Queirós P.J.P., Araújo J.P. (2019). Humanitude in the Humanization of Elderly Care: Experience Reports in a Health Service. Rev. Bras. De Enferm..

[32] Ribeiro J.P., Gomes G.C., Thofehrn M.B. (2014). Health Facility Environment as Humanization Strategy Care in the Pediatric Unit: Systematic Review. Rev. Da Esc. De Enferm. Da USP.

[33] Pienaar L., Reynolds F. (2015). “A respite thing”: A qualitative study of a creative arts leisure programme for family caregivers of people with dementia. Health Psychol. Open.

[34] Chiang M., Reid-Varley W.B., Fan X. (2019). Creative art therapy for mental illness. Psychiatry Res..

[35] Gaiha S.M., Salisbury T.T., Usmani S., Koschorke M., Raman U., Petticrew M. (2021). Effectiveness of arts interventions to reduce mental-health-related stigma among youth: A systematic review and meta-analysis. BMC Psychiatry.

[36] Staricoff R.L. (2006). Arts in health: The value of evaluation. J. R. Soc. Promot. Health.

[37] Kinsella E.A., Vanstone M. (2010). An international conference on engaging reflection in health professional education and practice: Emerging conversations on the arts in health and social care. Reflective Pract..

